# Vancomycin-induced linear IgA bullous dermatosis in a patient with cancer

**DOI:** 10.1016/j.idcr.2022.e01671

**Published:** 2022-12-22

**Authors:** Naoya Itoh, Nana Akazawa, Takafumi Yanaidani, Waki Hosoda, Mayumi Mori

**Affiliations:** a Division of Infectious Diseases, Aichi Cancer Center Hospital, 1-1 Kanokoden, Chikusa-ku, Nagoya, Aichi 464-8681, Japan; b Collaborative Chairs Emerging and Reemerging Infectious Diseases, National Center for Global Health and Medicine, Graduate School of Medicine, Tohoku University, 2-1 Seiryo-machi, Aoba-ku, Sendai, Miyagi 980-8575, Japan; c Department of Gastroenterology, Aichi Cancer Center Hospital, 1-1 Kanokoden, Chikusa-ku, Nagoya, Aichi 464-8681, Japan; d Department of Pathology and Molecular Diagnostics, Aichi Cancer Center Hospital, 1-1 Kanokoden, Chikusa-ku, Nagoya, Aichi 464-8681, Japan; e Department of Dermatology, Aichi Cancer Center Hospital, 1-1 Kanokoden, Chikusa-ku, Nagoya, Aichi 464-8681, Japan

A 72-year-old Japanese woman with advanced cystic duct carcinoma presented with bullous lesions on the palms and soles, and erythema of the trunk, back, and extremities. She was treated with gemcitabine and cisplatin for approximately 7 months. Her last chemotherapy session had been performed nearly 1 month prior. She underwent an endoscopic ultrasound-guided hepaticogastrostomy (EUS-HGS) for obstructive jaundice caused by tumor obstruction and subsequently developed cholangitis. Treatment with intravenous sulbactam-cefoperazone 2 g/day was initiated. Four days after initiation of antimicrobial therapy, intravenous vancomycin was administered because methicillin-resistant Staphylococcus aureus was isolated in bile culture; administration was initiated at a loading dose of 1000 mg, followed by 500 mg every 12 h. Seven days after vancomycin administration began, painful bullous lesions appeared on the bilateral palms, and erythema appeared on the trunk, followed by rapidly progressive bullae and erythema on the back, extremities, and soles of the feet. Physical examination revealed large tense bullae on both palms (Fig. 1a) and soles, and circular erythema on the extremities bilaterally and on the abdomen and back (Fig. 1b). Differential diagnoses included disseminated varicella-zoster virus infection, bullous pemphigoid, and linear IgA bullous dermatosis (LABD). Skin biopsies were performed from the palms and soles, and histopathologic examination revealed that superficial layers of the dermis had exudates and infiltration of inflammatory cells, predominantly neutrophils (Fig. 1c). Direct immunofluorescence showed IgA deposition along the dermo-epidermal junction (Fig. 1d). Serum anti-BP180 antibody was negative, serum varicella zoster virus (VZV)-IgG was positive, and VZV-IgM was negative. After 11 days of vancomycin administration, vancomycin-induced LABD was suspected, and vancomycin was discontinued while sulbactam-cefoperazone was continued. Three days after discontinuation of vancomycin, the lesions started to resolve. Sulbactam-cefoperazone was given for a total of 14 days. At follow-up, approximately one month later, complete disappearance of the skin lesions was observed.

**Fig. 1:**
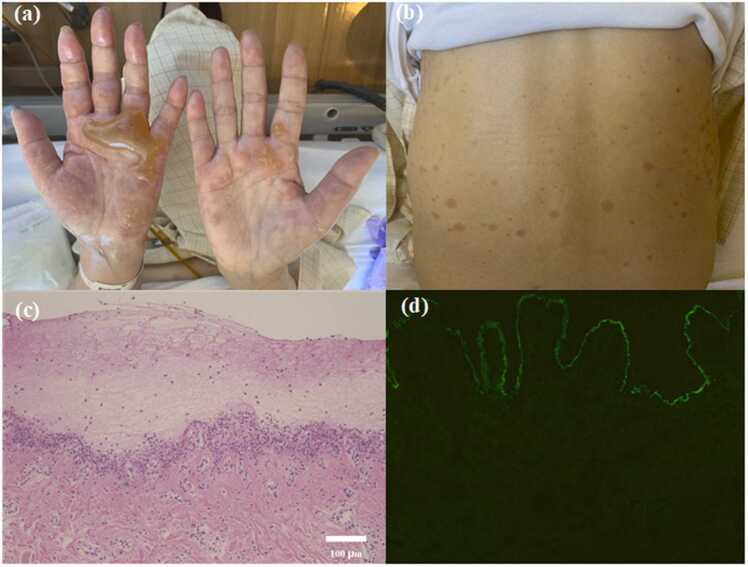
(a) Bullae on both palms. (b) Circular erythema on the back. (c) Histology of the biopsy specimen of a blister displaying an erosion with an exudate and marked neutrophil infiltration, consistent findings with a subepidermal blister. (H&E stain, ×100). (d) Direct immunofluorescence shows IgA deposition along the dermo-epidermal junction.

LABD is a rare autoimmune bullous disease characterized by linear deposition of IgA at the dermo-epidermal junction [1]. Drugs, autoimmune diseases, infections, and malignancies are possible contributors to LABD [2]. Many drugs can cause LABD, but vancomycin is the most frequently reported causative agent [3], [4]. Early diagnosis is necessary for the management of LABD since the discontinuation of the causative agent is vital.

## Author contributions

NI was involved in the literature review, planning, and writing of the manuscript. NI, NA, TY, and MM were involved in patient care. WH conducted the laboratory analyses. All the authors interpreted the data, drafted, and critically revised the manuscript, and approved the final version.

## CRediT authorship contribution statement

**Naoya Itoh**: Conceptualization, Writing – original draft, Funding acquisition. **Nana Akazawa**: Writing – review & editing. **Takafumi Yanaidani**: Writing – review & editing. **Waki Hosoda**: Writing – review & editing. **Mayumi Mori**: Writing – review & editing.

## Declaration of interest

None.

## Data Availability

All the relevant data are contained in the report.
